# The Association between Shift Work and Health-Related Productivity Loss due to Either Sickness Absence or Reduced Performance at Work: A Cross-Sectional Study of Korea

**DOI:** 10.3390/ijerph17228493

**Published:** 2020-11-16

**Authors:** Seong-Sik Cho, Dong-Wook Lee, Mo-Yeol Kang

**Affiliations:** 1 Department of Occupational and Environmental Medicine, Dong-A University College of Medicine, Busan 49201, Korea; 0361pt@hanmail.net; 2 Department of Preventive Medicine, Seoul National University College of Medicine, Seoul 03080, Korea; taesanglee89@gmail.com; 3 Department of Occupational and Environmental Medicine, Seoul St. Mary’s Hospital, College of Medicine, The Catholic University of Korea, Seoul 06591, Korea

**Keywords:** productivity loss, presenteeism, absenteeism, shift work, gender difference

## Abstract

*Background*: The purpose of the present study was to investigate the association between shift work and health-related productivity loss (HRPL) due to either sickness absence or reduced performance at work. *Methods*: From January 2020 to February 2020, data were collected using the web-based questionnaire. Workers in Korea (*n* = 4197) were selected with the convenience sampling method. To evaluate HRPL, the Korean version of the “Work Productivity and Activity Impairment Questionnaire” was used. The nonparametric association between shift work and HRPL was determined. To estimate productivity loss by shift work, generalised linear models were used, and the productivity loss of workers who did not do shift work was used as the reference. Contrasts between the reference (non-shift work) and shift work, including the shift work subtype, were demonstrated. In the adjusted model, age, gender, and occupation were included as covariates. To test whether there were differences in this association by gender, a gender-stratified analysis was conducted. *Results*: Shift work significantly reduced productivity (2.5% points; 95% CI: 0.2–4.6). The fixed night shift had the largest productivity loss (7.7% points; 95% CI: 1.8–13.7), and the relationship between HRPL and shift work was more prominent among female workers. *Conclusions*: Shift work is related to an increase in HRPL, and there are gender differences in this association. Our study further indicated that a fixed night shift is most detrimental to workers’ health and productivity.

## 1. Introduction

Shift work has become an increasingly widespread practice across a variety of occupations. Consequently, it has been estimated that almost 15–30% of the workforce in industrialised countries is involved in shift work [1]. Shift work is defined as a working-time arrangement in which the working hours in the workplace are successive to each other, so that the working hours exceed those of the individual workers [2]. There are many atypical work schedules called shift work, which include fixed evenings, fixed nights, irregular shifts, and rotating shifts.

A major reason for shift work is that modern technological and organisational advances have made it possible to perform many activities at any time of the day or night [3]. This “24-h society” requires that important services be provided at all times. The industry has to operate 24 h a day as the production process is much longer than 8 h and needs to be carried out continuously. Other manufacturing industries use expensive machines that must operate continuously to remain profitable. However, there is clear evidence that shifts and night work that are often enforced for economic reasons to maximise the use of costly equipment and increase productivity can present high human costs [3].

Previous research has demonstrated a variety of negative biological, psychological, and social effects of shift work and other atypical work schedules on workers’ health. Shift work, particularly the night or rotating shift, is considered a serious work stress factor and has adverse health effects such as sleep problems, depression, cancer, diabetes, hypertension, and cardiovascular disease [4,5]. It is also an important factor that causes human error and consequent work accidents and injuries [6].

As a result, shift work has also been linked to an increased risk of sick leave [7,8,9,10], which may thus seriously undermine any potential economic benefit derived by introducing it. In a systematic review of the relationship between shift work and sickness absence, a strong support for the relationship between fixed evening shifts and long sick leaves among female healthcare workers has been indicated [11]. However, there has been limited research on the extent of sickness presenteeism or reduced performance at work compared to sickness absence, even though sickness presenteeism may occur more frequently and may be much costlier than sickness absenteeism. Therefore, the purpose of the present study was to investigate the association between shift work and health-related productivity loss (HRPL) due to either sickness absence or reduced performance at work. In addition, this study examined the effect of gender, age, and shift schedule types as potential moderators of the relationship between shift work and HRPL.

## 2. Materials and Methods

### 2.1. Study Design and Participants

This study was a cross-sectional study on the association between shift work and HRPL. The target population was workers in Korea, 19 years of age or older. Data were collected from a web-based questionnaire by Panelnow (https://www.panelnow.co.kr). The survey was carried out through an online panel survey service (Data Spring Korea Inc., Seoul, Korea) and study participants were recruited online. A total of 4197 participants, who agreed to participate in the survey, completed questionnaires. The study included employees (*n =* 3890) and self-employed individuals (*n =* 307). The online survey system enabled all participants to complete the questionnaire. With the aid of this survey system, when participants answered all questions, the survey could be completed; for this reason, data were collected without missing variables. For participants to complete the questionnaire on the internet, ten minutes were required. However, 51 participants who answered that their work schedules were other types were excluded from the final analysis. 

### 2.2. Ethical Considerations 

The present study was approved by the Institutional Review Board (IRB) of Seoul Saint Mary’s Hospital (KIRB-20200219-014).

### 2.3. Measurement of Health-Related Productivity Loss

Structured questionnaires were frequently used to measure work productivity loss. Several questionnaires have been used to estimate absenteeism and presenteeism caused by health problems on paid working days [12]. To evaluate HRPL, the “Work Productivity and Activity Impairment Questionnaire: General Health version’ (WPAI:GH) was used. The WPAI:GH is composed of six items and can estimate absenteeism, presenteeism, and work productivity loss due to health problems. Absenteeism is defined as the percentage of work time missed in the past seven days due to health problems, and was calculated as [hours lost from work because of health problems in the last week/(lost hours due to health problems + working hours in the last week)]. Presenteeism is defined as the percentage of impairment experienced at work in the past 7 days owing to health problems; it was calculated as a percentage of the productivity loss due to health problems in the last week. The percentage of overall HRPL was calculated using the formula absenteeism + presenteeism × hours worked in the last week. The reliability and validity of WPAI:GH was tested in a previous study [13]. The Korean version is available on the internet and harmonization of the translation process consists of independent translations, back-translation, and expert review [14]. 

### 2.4. Measurement of Shift Work, Types of Shift Schedule, and Other Covariates

The shift work status (no vs. yes) and various shift schedule types (fixed evening, fixed night, rotating shift, 24 h shift, split shift, and irregular shift) were surveyed using questionnaires.

Other information on age, gender, level of education (≤High school/college, university, or graduate school), marital status (single, married, separated or widowed, or divorced), employment status (precarious or non-precarious), occupation (professional, clerical, service and sales, manual, smoking habits (current smoker or non-smoker), binge drinking (drinking 7 units of alcohol or more at a time), and exercise (moderate- or high-intensity training) were assessed using questionnaires.

### 2.5. Statistical Analysis

The data of 4146 workers, including self-employed workers, were used in the final analysis. The general and occupational characteristics of the study population were described by shift work. The numbers and percentages were present, and Chi-square test was used to assess basal characteristic differences between workers who did shift work and those who did not. To estimate productivity loss by shift work and various shift schedules, a generalised linear model was used and the productivity loss of employees who did not do shift work was used as the reference group. Contrasts between the reference (non-shift work) and shift work, including the shift work subtype, were demonstrated. In the adjusted model, age, gender, and occupation were included as covariates. To test whether there were differences in productivity loss between genders, a gender-stratified analysis was conducted. Finally, to examine differences in productivity loss among different age groups (20–29, 20–39, 40–49, 50 or older), age-group-stratified analysis was performed. All statistical analyses were carried out using Stata version 16.1 (Stata company, College Station, TX, USA), and *p-*value < 0.05 was considered to be statistically significant.

## 3. Results

Table 1 shows the characteristics of the study participants. There was a total of 4146 participants, composed of 2098 (50.6%) males and 2048 (49.4%) females. A higher percentage of male workers (54.1%) were involved in shift work, compared to female workers. There was a tendency for younger participants to do shift work. A higher proportion of participants engaged in shift work was unmarried. Shift work is associated with education, occupation, employment status, income level, and weekly working hours. Participants with a lower educational level and a lower income level were linked to shift work. The higher proportion of blue-collar workers (48.6% and 23.5% for service and sales workers, and manual workers, respectively) did shift work. Precarious employment was also linked to shift work. Shift workers worked longer hours, and shift work was associated with binge drinking.

**Figure 1:**
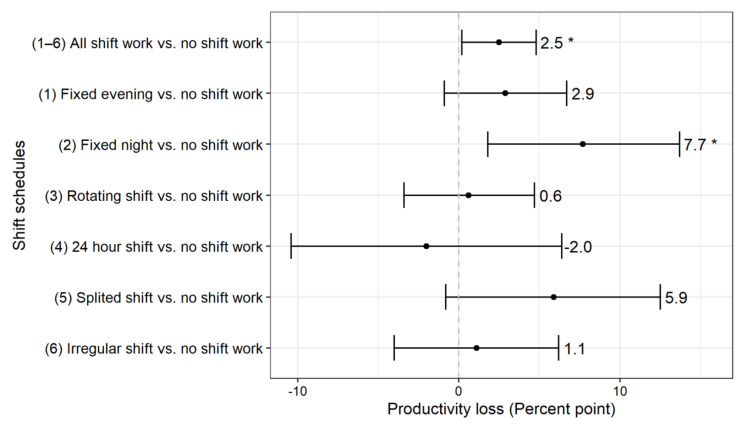
Adjusted differences in work productivity impairment according to shift work schedules; * *p*-value < 0.05.

Table 2 shows productivity loss brought upon by the shift work and shift schedule. Shift work significantly reduced productivity in both the unadjusted model (2.9% points; 95% CI: 0.7–5) and the adjusted model (2.5% points; 95% CI: 0.2–4.6). Absenteeism, rather than presenteeism, contributed to the differences in productivity loss. When it came to various subtypes’ schedule of shift work, the fixed evening, fixed night, and split shift were linked to more reduced productivity. In the adjusted model, fixed night work showed 7.7% points reduced productivity (95% CI: 1.8–13.7); the other subtypes’ schedule of shift work did not show significant productivity loss compared to reference (Figure 1).

Table 3 presents the gender-stratified productivity loss ascribed by shift work and various subtypes of shift schedules. The results of Table 3 were made using the adjusted model, and age and occupation were included. Females were more prone to productivity loss brought upon by shift work than males. When we observed the subtypes of shift work, males’ productivity loss was linked to fixed night and females’ productivity loss was linked to split shift.

The appendix table shows the age-group-stratified productivity loss due to shift work. We could not observe any significant differences in productivity loss by shift work among the age groups. 

## 4. Discussion

The results indicated that shift work is associated with increased HRPL. The associations were complex and differed by gender and type of shift schedule. The fixed night shift has the largest productivity loss. The relationship between HRPL and shift work was more prominent among female workers. In line with our findings, there has been considerable evidence for a negative relationship between shift work and sickness absence, especially for fixed night shifts [7,9]. For example, a recent longitudinal multilevel study at a Norwegian hospital also showed a clear relationship between shift work and increased risk of sickness absence. Relationships were maintained regardless of gender, age group, and whether or not the individuals had children [10]. However, some studies have found no significant relationship between shift work and sickness absence [15,16]. 

The negative effects of shift work on health are primarily due to disturbances in the normal circadian rhythm (e.g., sleep–wake cycle, hormones such as melatonin and cortisol, attention, performance, body temperature, and metabolism). The circadian rhythm is a major body rhythm, and many systems in the body are active at certain times of the day and are inactive at all others. People perform best when arousal and internal physical activity are high and worst when arousal and activity are low. When working the night shift, one should work when his or her circadian rhythm is low and sleep when it is high. These schedules mean that workers try to stay alert when their circadian rhythm is low. This is a poorer schedule for workers’ health, compared to typical day work schedules, which means it is disadvantageous for work performance [3]. If a worker has also lost sleep, fatigue and sleepiness could combine with the circadian low point to worsen the worker’s health condition and ability to perform. Poor performance is sometimes a cause of errors that could lead to accidents or injuries, making productivity loss greater. A meta-analysis to quantitatively assess the effects of fixed and rotating shift work schedules on sleep length suggested that fixed night shifts resulted in a decrease, whereas fixed evening shifts resulted in an increase in sleep length [17]. This could explain why the highest HRPL is found in fixed night workers, as compared to day workers. Studies with physiological measures would be helpful to understand the underlying mechanism in terms of disturbances of the normal circadian rhythm of bodily functions.

Another reason why fixed night workers are the most vulnerable to HRPL is their withdrawal from social relationships, including those with their family and friends [3]. Because of the opposite life patterns, overnight workers have little chance to maintain social relations; thus, this obviously increases the risk of social isolation. Having a different lifestyle pattern compared to the rest of their family and friends can reduce the intensity of social–family support as a result of limited interaction [9,18]. In addition, difficulty engaging in leisure activities and voluntary organisation activities might have negative effects on workers’ well-being and mental health. This social–family problem, in addition to the lack of sleep and signs of irritability, fatigue, and mood swings, can lead to HRPL.

There has been inconclusive evidence to show that there is an association between “rotating” shift work and HRPL. In accordance with our findings, several studies did not find a link between rotating shift work and sick leave [9,19]. Other studies reported a higher prevalence of sick leave among workers with 3- or 2-shift schedules [20,21,22]. These differences may be due to the adjustment for job characteristics, sample size, and the effect of selection bias. Therefore, future studies with larger sample sizes and prospective designs that take into account potential confounders, mediators, and effect modifiers will be valuable and can provide a better understanding of the likely causal pathways between shift work and HRPL.

We found a statistically significant positive relationship between shift work and HRPL in women but not in men. The present results support gender’s moderating effect on the negative relationship between shift work and HRPL. Findings from previous studies also show that female shift workers had a larger increase in sickness absences than males [10]. One possible explanation for this is that gender differences occurred because women responded more severely to shift work than men did (e.g., because of biological or social differences, or differences in the type of job they held). For example, female workers may suffer severely from hormone disruption as a result of shift work. Melatonin is a hormone that maintains one’s circadian rhythm and helps regulate other hormones such as female sex hormones; thus, disruption of the circadian rhythm can disturb the endocrine system [23]. In addition, it is important to recognise that having children could be a moderating factor on the relationship between shift work and HRPL [10] and that, in East Asian cultures, primary responsibility is commonly placed on mothers. Thus, women might have experienced higher levels of work–family conflict from shift work [24].

Contrary to expectations, stratified analysis by age group found that age had no moderating effect. In authors’ experience, older workers are less capable of shift work because of the decreased ability to recover from fatigue that comes with aging. Aging is known to slow down circadian adaptation to night shifts and increases the risk of sleep disturbances and harmful health effects [25]. Although the present study could not find a consistent dose–response relationship by age group (Appendix A), it should be recognised that selection bias could not be ruled out and might have affected the results of this study. This is because the shift workers included in the present study can represent a relatively healthy portion of the workforce. Participants in this study were likely to have already adapted to their work schedule prior to the survey. Hence, survey participants still engaged in shift work may be considered as shift “survivor” and this “healthy shift worker survivor effect” may lead to biased outcomes. Therefore, the true impact of shift work on HRPL would be underestimated in this cross-sectional analysis. The selection process is considered an important methodological issue related to shift work research.

Additionally, several other limitations should be considered when interpreting the results of the present study. First, the cross-sectional design of the study does not permit further explanation of the causal relationship. Second, the data collection was based on convenience samples. Therefore, the sample estimate may not reflect the actual effect among the target populations, and we cannot argue that the results of the present study are representative of or generalizable to other populations. Third, although self-reported data on HRPL are usually considered reliable and valid [12], the measurement of the impairment of one’s work performance is still a major challenge for research. Finally, the method of the HRPL assessment in the current study did not permit us to differentiate between specific health problems, although this would have been useful for the development of a preventive strategy.

## 5. Conclusions

The findings of the present study suggest that shift work is related to an increase in HRPL among the Korean working population, and that there are gender differences in this association. The results further indicate that a fixed night shift is most detrimental to workers’ health and productivity. Although shift work was introduced for purely economic reasons, it could impair workers’ performance efficiency and increase productivity loss if it is applied inappropriately. By investigating the effects of gender and types of shift work, the present study may contribute to the development of interventions for productivity loss prevention. However, there are still a lot of unanswered questions about how this could be achieved. The problem of shift work is complex, and any intervention must carefully consider several interrelated factors. Hence, more research is warranted in those areas.

## Figures and Tables

**Table 1:** The general and occupational characteristics of study population by shift work.

Characteristics	No Shift Work		Shift Work		
	*n*	%	*n*	%	*p*-Value *
Gender					0.046
Male	1727	49.91	371	54.08	
Female	1733	50.09	315	45.92	
Age					<0.001
20–29	742	21.45	215	31.34	
30–39	985	28.47	157	22.89	
40–49	1026	29.65	188	27.41	
50–59	499	14.42	92	13.41	
≥60	208	6.01	34	4.96	
Marital status					<0.001
Unmarried	1539	44.48	405	59.04	
married	1758	50.81	255	37.17	
divorced, widowed, other	163	4.71	26	3.79	
Education					<0.001
High school or less	625	18.06	197	28.72	
University, college	2515	72.69	462	67.35	
Graduate	320	9.25	27	3.94	
Occupation					<0.001
Managerial	444	12.83	59	8.6	
Clerical	1948	56.3	133	19.39	
Sales or service	597	17.25	333	48.54	
Manual	471	13.61	161	23.47	
Employment					<0.001
Non-precarious	2752	84.62	384	64.21	
Precarious	500	15.38	214	35.79	
Income quintile					<0.001
Lowest	816	23.58	226	32.94	
Lower middle	1075	31.07	213	31.05	
Upper middle	645	18.64	134	19.53	
Highest	924	26.71	113	16.47	
Working hours					<0.001
≤34	331	9.57	186	27.11	
35–40	1512	43.7	188	27.41	
41–52	1305	37.72	223	32.51	
≥53	312	9.02	89	12.97	
Smoking					0.491
Non-smoking	927	61.55	218	63.56	
Current smoking	579	38.45	125	36.44	
Binge drinking					0.022
No	1890	54.62	342	49.85	
Yes	1570	45.38	344	50.15	
Exercise					0.535
No	1786	51.62	363	52.92	
Yes	1674	48.38	323	47.08	

Note: *—*p*-value was estimated by Chi-square test.

**Table 2:** Health-related productivity loss (HRPL) due to shift work and various shift schedules in the Korean working population (Unit: percent point).

Work Schedule	Productivity Loss			Productivity Loss Due to Absenteeism			Productivity Loss Due to Presenteeism		
No Shift Work	ref	95% CI		ref	95% CI		ref	95% CI	
Shift work	2.5	0.2	4.8	0.8	0.1	1.5	1.7	−0.5	3.9
Shift schedules									
Fixed evening	2.9	−0.9	6.7	1.0	−0.2	2.2	1.9	−1.7	5.4
Fixed night	7.7	1.8	13.7	2.7	0.8	4.5	5.0	−0.5	10.6
Rotating shift	0.6	−3.4	4.7	0.2	−1.1	1.5	0.4	−3.4	4.2
24 h shift	−2.0	−10.4	6.4	−0.9	−3.5	1.7	−1.1	−8.9	6.8
Split shift	5.9	−0.8	12.5	3.1	1.0	5.2	2.8	−3.4	9.0
Irregular shift	1.1	−4.0	6.2	−0.5	−2.1	1.1	1.6	−3.2	6.4

Estimated by generalized linear model and contrast to reference (no shift work). Analytic model was adjusted by age, sex, and occupations.

**Table 3:** Gender-stratified health-related productivity loss (HRPL) due to shift work and various shift schedules in the Korean working population (Unit: percent point).

Work Schedule	Male			Female		
No Shift Work	Ref	95% CI		Ref	95% CI	
Shift Work	0.6	−2.4	3.7	4.7	1.2	8.2
Shift Schedules						
Fixed evening	3.0	−2.1	9.7	2.4	−2.7	7.4
Fixed night	7.7	0.5	14.7	6.7	−3.8	17.2
Rotating shift	−3,4	−8.0	1.2	11.1	3.2	19.0
24 h shift	−1.8	−11.9	8.3	−2.9	−17.3	11.6
Split shift	−0.2	−11.8	11.4	9.2	1.0	17.5
Irregular shift	1.2	−5.5	7.8	1.2	−6.6	9.0

Estimated by generalized linear model and contrast to reference (no shift work); analytic model was adjusted by age and occupations.

## Appendix A

**Table A1:** Age-group-stratified productivity loss due to shift work (Unit: percent point).

Age Group		Productivity Loss			Productivity Loss due to Absenteeism			Productivity Loss due to Presenteeism		
20–29	Shift work									
	(−)	ref	95% CI		ref	95% CI		ref	95% CI	
	(+)	3.5	−1.1	8.1	1.4	−0.1	2.9	2.1	−2.2	6.4
30–39	Shift work									
	(−)	ref	95% CI		ref	95% CI		ref	95% CI	
	(+)	−0.0	−4.8	4.8	1.0	−0.2	2.2	−1.0	−5.6	3.6
40–49	Shift work									
	(−)	ref	95% CI		ref	95% CI		ref	95% CI	
	(+)	4.3	−0.0	8.7	0.8	−0.6	2.3	3.5	−0.5	7.5
≥50	Shift work									
	(−)	Ref	95% CI		ref	95% CI		ref	95% CI	
	(+)	2.1	−2.8	7.0	−0.3	−2.1	1.4	2.4	−2.0	6.9

Estimated by generalized linear model and contrast to reference (no shift work); analytic model was adjusted by sex, occupations.
